# Comparison of postoperative recovery of patients who underwent laparoscopic-assisted radical resection of right colon cancer with modified triangular anastomosis or tubular anastomosis: a retrospective cohort study

**DOI:** 10.1186/s12893-021-01086-6

**Published:** 2021-02-10

**Authors:** Tianfang Xia, Zhenguo Pan, Jie Zhang, Guo Xu

**Affiliations:** 1 Department of General Surgery, The Affiliated Huaian No.1 People’s Hospital of Nanjing Medical University, No. 6 Beijing West Road, Huaiyin, Huaian, 223300 Jiangsu China; 2 Department of Gastroenterology, The Affiliated Huaian No.1 People’s Hospital of Nanjing Medical University, Huaian, China

**Keywords:** Modified triangular anastomosis, Tubular anastomosis, Laparoscopic-assisted radical resection, Right colon cancer

## Abstract

**Background:**

We compared the advantages and disadvantages of modified triangular anastomosis and tubular anastomosis for digestive tract reconstruction in patients undergoing laparoscopic-assisted radical resection of right colon cancer.

**Methods:**

This was a retrospective cohort analysis of 92 cases of laparoscopic-assisted resection of right colon cancer, treated from June 2017 to June 2018, at the Huai’an No. 1 People’s Hospital in China. Patients were divided into a modified triangular anastomosis group (n = 33) and a tubular anastomosis group (n = 59). In the modified triangular anastomosis group, digestive tract reconstruction was conducted using side-to-side anastomosis of the ileo-transverse colon with a 60-mm linear stapler. The common entry hole was closed with a running suture. The tubular anastomosis group underwent end-to-side anastomosis of the ileo-transverse colon with a tubular stapler anchor placed at the end of the ileum.

**Results:**

At baseline and perioperatively, there were no significant between-group differences in age, sex, body mass index, tumor location, pathological stage, or tumour size (P > 0.05). There were also no significant between-group differences in operation time, estimated blood loss, the number of harvested lymph nodes, the first postoperative flatulence time, hospitalisation time, or postoperative complications (P > 0.05); however, the total cost of hospitalization for the triangular anastomosis group was significantly lower than the tubular anastomosis group (P < 0.05).

**Conclusion:**

Modified triangular anastomosis is a safe and feasible procedure for laparoscopic-assisted radical resection of right colon cancer. These results affirm the safety and effectiveness of total laparoscopic radical resection of right colon cancer. Given the equivalent outcomes between the two procedures, the modified triangular procedure may be more a more cost-effective option for clinical application.

## Background

Similar to complete mesocolic excision (CME) and central vascular ligation (CVL), the success rate of laparoscopic radical resection of right colon cancer has greatly improved [1–4], with reduced complications and widespread confirmation of the procedure’s safety and feasibility. Laparoscopic radical resection has become the first choice for treating stage II, and some stage III, right colon cancers [5–7]. During laparoscopic surgery, digestive tract reconstruction is critical after complete mesocolon dissociation and tumour excision. At present, the main anastomotic methods are tubular stapler reconstruction and linear triangular anastomosis [8–11].

Recently, complete laparoscopic radical resection of right colon cancer has been widely applied. Its advantages include a smaller abdominal incision, lesser trauma, and faster recovery [12, 13]. Comparing laparoscopic-assisted and total laparoscopic radical resection of colon cancer, both tubular and triangular anastomoses are feasible approaches. Triangular anastomosis is commonly used during total laparoscopic surgery, and includes a modified triangular anastomosis and overlapping triangular anastomosis procedures [14]. In the modified triangular anastomosis, after excision of the lesion, a small puncture is made on the antimesenteric wall of the ileum and colon respectively. Then, a side-to-side anastomosis is created with a 60-mm disposable linear cutting stapler, the common entry hole is closed using a running suture [15]. For the tubular anastomosis manoeuvre, the anvil of a circular stapler is positioned in the lumen of the distal ileum using a purse string suture. The device is inserted through the open end of the colon, after which the trocar and anvil are connected. The instrument is closed, fired, opened and carefully withdrawn. The open end of the colon is now closed by the linear stapler [16].

The differences between tubular anastomosis and triangular anastomosis—especially operation time, bleeding loss, number of harvest lymph nodes and the incidence of serious postoperative complications such as anastomotic leakage—determine the effects of surgical treatment. Consequently, the best procedure should be selected.

We retrospectively analysed the clinical data of 92 cases of laparoscopic-assisted radical resection of right colon cancer that occurred at the Affiliated Huai'an No. 1 People's Hospital of Nanjing Medical University from June 2017 to June 2018. During this period, there were 59 cases of tubular anastomosis and 33 cases of modified triangular anastomosis. We comparatively assessed the safety and effectiveness of these two approaches for total laparoscopic radical resection of right colon cancer.

## Methods

### Subjects

Included patients were older than 18 years with stage I-III resectable right hemicolon adenocarcinoma (AJCC-8th TNM-staging system), confirmed by preoperative imaging and colonoscope biopsy. All patients had tumours located in the ileocecal, ascending colon, or hepatic flexure of the colon. We excluded patients without complete clinical data, patients with double or multiple primary colorectal tumours, and patients with intestinal obstruction. We also excluded patients with severe heart disease, lung disease, severe liver or kidney insufficiency, severe diabetes mellitus, or severe neurological diseases. Lastly, we excluded patients with unresectable distant metastases and those with extensive intraperitoneal adhesions.

After selection according to the above criteria, the clinical data of 92 patients—from admission to discharge—were retrospectively analysed. Among them, 33 patients underwent a modified triangular anastomosis in the digestive tract and 59 patients underwent tubular anastomosis. The same physicians operated on the patients. All patients underwent the same preoperative examinations, including routine woodwork, blood biochemistry, tumour markers, chest and abdominal CT, enteroscopy, and biopsy. All patients and their families were informed consented to all procedures.

### Surgical methods

**Fig. 1:**
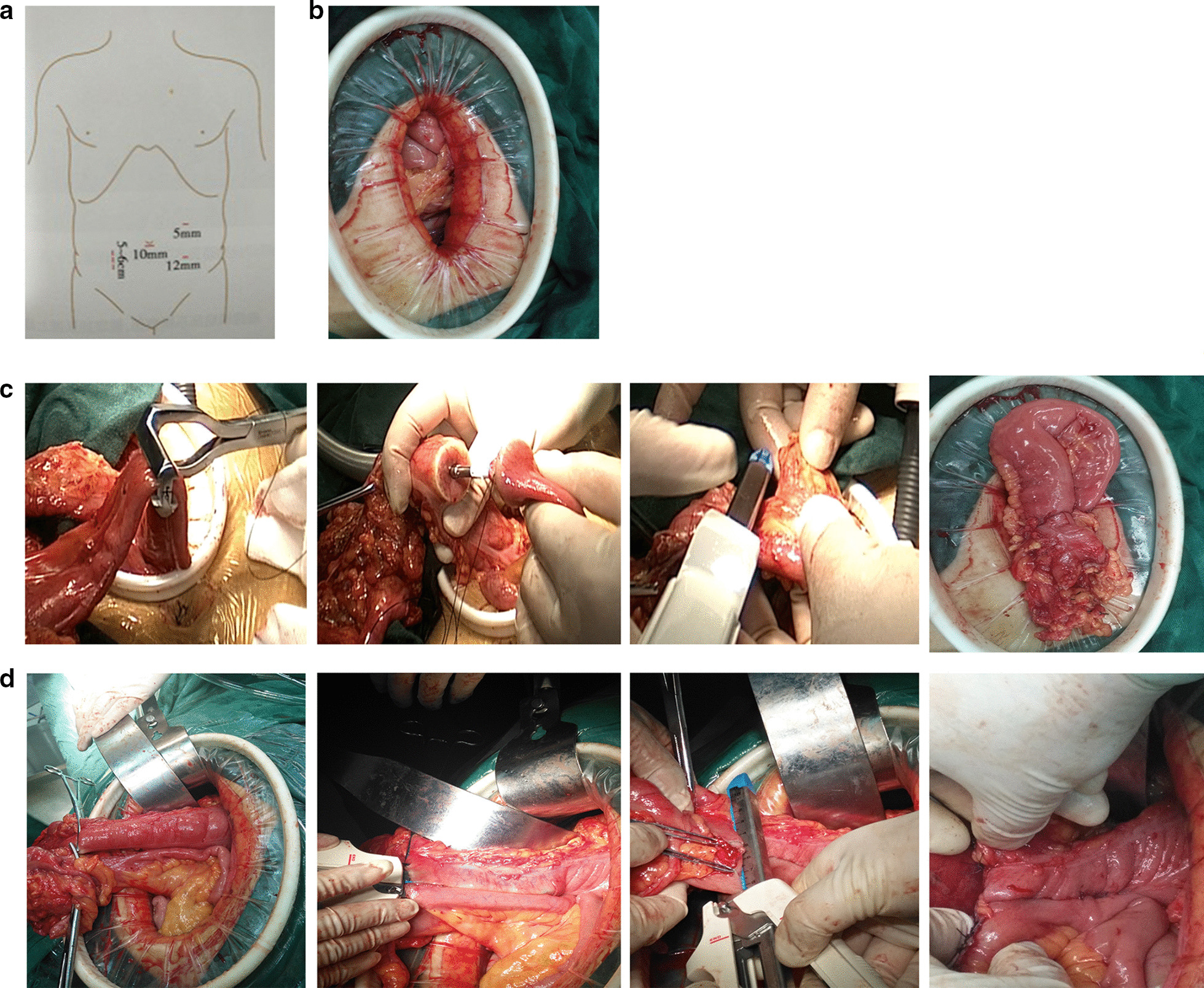
Resection and reconstruction of the digestive tract in laparoscopic-assisted radical resection of right colon cancer. **a** A 5–6 cm incision is made at the right upper abdomen and a lap-protector is used to protect the skin. **b** An end-to-side anastomosis of ileo-transverse colon is conducted using a tubular anastomosis. **c**, **d** Side-to-side anastomosis of the ileo-transverse colon is conducted using a modified triangular anastomosis

1. *Arrangement of operative position and surgical access.* After tracheal intubation under general anaesthesia, each patient was placed in the left oblique lateral position with his or her head-down. Surgical access was achieved using the four-hole method. A 10 mm incision was made below the umbilicus and used for observation. After this, an artificial pneumoperitoneum was established, and the pneumoperitoneum pressure was maintained at 13–15 mmHg. The abdominal cavity was explored to determine the lesion location. A 12 mm Trocar at the lateral edge of the left rectus abdominis was used as the main surgical access point, and a 5 mm Trocar at the head was used for accessory surgical access. At the right collarbone midline, a 5 mm Trocar was placed at the umbilicus level, which was expanded to 5–6 cm at the later stage to take the specimen (Fig. 1a).
2. *Anatomy of the intestinal duct and mesentery according to CME.* The tail-dorsal approach was used and the small intestine was pulled to the head side and fixed with laparoscopic gauze. The mesentery of the small intestine was held and stretched to the head side. The peritoneum of the mesenteric attachment was incised from the ileocecal valve to the duodenum. Next, the posterior peritoneum, anterior renal fascia, and the posterior mesenteric space were freed from the anterior ventral duodenum to the anterior part of the duodenum and the posterior head of the pancreas. Then, gauze was placed as a marker to protect the duodenum. The patient was placed in a horizontal position, and the operator pushed the small intestine to the tail side, lifted the transverse colon to the head side, lifted the ileocolic artery and ileocolon to the ventral side and cut the mesentery to achieve penetration with the free layer of the anterior–posterior peritoneal region. After confirming the gauze marker, the vessels of the ileocolon, right colon, and middle colon were ligated. After the treatment of these vessels, the operator pulled down the transverse colon and greater omentum and entered the omental sac. The right gastroepiploic artery and vein were cut off, the gastrocolic ligament, the hepatocolic ligament, and the right peritoneum were dissociated to complete dissociation of the intestinal tract and mesentery for removal.
3. *Resection of intestinal tract and reconstruction of the digestive tract.* A 5–6 cm incision through the right upper abdomen was made through the rectus abdominis. Then, the abdominal wall layers were cut to enter the abdominal cavity, and a lap-protector was used to protect the skin. We used two linear cutting closers (Tianchen LC8038) cut 15 cm from the end of the ileum and the right hemi-transverse colon (10 cm from the distal part of the tumour) respectively, and the specimen was taken out of the body.
  1. Tubular anastomosis. We opened the end of the ileum and and sterilized the intestinal tract by iodophor gauze, a tubular stapler anchor(Johnson CCS25) was placed at the end of the ileum, then we implanted the tubular stapler from the end of the transverse colon, rotated the connecting rod 5–7 cm from the end of transection to the mesenteric margin and docked with the stapler anchor, After examining the anastomotic site patency and confirming that there was no bleeding, the broken colon end was closed using a linear incision closer (Tianchen LC8038). The anastomotic site and the broken end were sutured tightly with absorbable sutures (Fig. 1c).
  2. *Modified triangular anastomosis. *A side-to-side anastomosis of the ileo-transverse colon was conducted. The intestinal tract located 8 cm from the end of the transverse colon and the end of the ileum was sutured using one needle. The antimesenteric wall of the ileum and colon was perforated. Iodophor gauze was used to disinfect the intestinal tract. Then, a side to side anastomosis at the antimesenteric border of the ileum and colon was performed by inserting a cutting closer (Frankenman HJQ 80 × 4.5). After the anastomosis was unobstructed without bleeding, a linear cutting closer (Tianchen LC8038) was used to close the common opening (Fig. 1d).
4. *Abdominal closure.* The auxiliary incision was closed after checking the instrument correctly and the pneumoperitoneum was reconstructed. After confirming that the mesentery at the reconstructive anastomosis was not twisted and that the drainage tube was indwelling, the abdomen was rinsed.

### Statistical analysis

SPSS 16.0 statistical software was used to analyse all data, and normally distributed data are expressed as (x ± S). Nominal data are expressed by frequency (percentage). We used *t*-tests to analyse operation time, intraoperative bleeding loss, the number of harvested lymph nodes, time to the first postoperative flatulence, hospitalisation duration, and total expenses. The incidence of postoperative complications was measured using the chi-square test. Significant between-group differences were indicated by P-values < 0.05.

## Results

The operation was successfully completed in all 92 patients. Thirty-three patients underwent modified triangular anastomosis of the digestive tract, with a mean age of (59 ± 2.4) years. Fifty-nine patients underwent tubular anastomosis, with a mean age of (60.5 ± 1.4) years. At baseline, there were no significant between-group differences in age, sex, body mass index, tumor location, pathological stage, or tumour size (P > 0.05; Table 1).

**Table 1:** The comparison of clinicopathological factors in the study cohort of 92 patients undergoing different anastomotic methods

	Modified triangular anastomosis (n = 33)	Tubular anastomosis (n = 59)	Statistical value	P value
Age (years)	59 ± 2.437	60.58 ± 1.437	t = 0.5956	0.5529
Gender (n; %)				
Male	20 (60.61%)	42 (71.19%)	^χ2^ = 1.078	0.299
Female	13 (39.39%)	17 (28.81%)		
Body mass index (kg/M^2^)	20.68 ± 0.24	20.29 ± 0.23	t = 1.09	0.27
Tumor location			^χ2^ = 2.113	0.348
Hepatic flexure	16	22		
Ascending colon	9	14		
Cecum	8	23		
Pathological stage (AJCC 8e)				
I	1	8	Z = − 1.877	0.061
II	16	32		
III	16	19		
Tumor size, mean ± SD, cm	5.333 ± 0.4063	4.866 ± 0.3197	t = 0.8907	0.3755

The intraoperative and postoperative outcomes of both groups are shown in Table 2. There was no significant between-group differences in operation time, bleeding loss, the number of harvest lymph nodes, time to the first postoperative flatulence, hospitalisation time, or postoperative complications (P > 0.05); However, the total cost of hospitalisation was significantly lower for the triangular anastomosis group (P < 0.05).

**Table 2:** The intraoperative and postoperative outcomes of the two groups

	Modified triangular anastomosis (n = 33)	Tubular anastomosis (n = 59)	Statistical value	P value
Operation time (min)	168.2 ± 5.765	172.7 ± 5.08	t = 0.5628	0.575
Estimated blood loss (ml)	154.5 ± 16.75	122.1 ± 9.02	t = 1.867	0.0652
No. of harvest LNs	17.97 ± 1.359	16.46 ± 0.9143	t = 0.9513	0.344
First postoperative exhaust time (days)	3.242 ± 0.1445	3.22 ± 0.1056	t = 0.1242	0.9014
Hospitalization time (days)	12.97 ± 0.5461	13.24 ± 0.4169	t = 0.3872	0.6995
Hospitalization expenses (RMB)	49,593 ± 1308	56,548 ± 1166	t = 3.777	0.0003*
Postoperative complications(n)				
Anaemia	6	6	^χ2^ = 8.011, 8	0.4324
Hypoproteinemia	7	12		
Incision infection	2	4		
Itestinal obstruction	0	1		
Pneumonia	0	2		
Abdominal abscess	0	1		
Chylous leakage	0	3		
Abdominal hemorrhage	0	2		
Anastomotic bleeding	1	0		

*LNs* lymph nodes

*p < 0.05

Adverse events in the modified triangular anastomosis group included anaemia (n = 6), hypoproteinemia (n = 7), incision infection (n = 2), and anastomotic bleeding (n = 1). In the tubular anastomosis group, adverse events included anaemia (n = 6), hypoproteinemia (n = 12), incision infection (n = 4), intestinal obstruction (n = 1), pneumonia (n = 2), abdominal abscess (n = 1), chylous leakage (n = 3), and abdominal hemorrhage (n = 2). There were no significant between-group differences in the rates of adverse events (P > 0.05). All complications, for both groups, resolved with conservative treatment.

## Discussion

Right colon cancer is a common malignant tumour of the digestive tract [17, 18]. With the increased popularization and application of laparoscopic techniques for digestive tract tumour surgery, laparoscopic radical resection is frequently used to treat right colon cancer. Notably, the procedure is associated with less trauma, a shorter operation time, less anatomical obstruction, thorough lymph node dissection, and faster recovery [19, 20]. During laparoscopic radical resection of right colon cancer, the main anastomotic methods are tubular stapler reconstruction and linear triangular anastomosis, the decision as to which the anastomosis method should be used depends on factors such as surgical quality and the occurrence of postoperative complications [21]. In this study, all patients underwent laparoscopic-assisted radical resection of right colon cancer. We retrospectively analysed the data of patients who underwent either tubular or triangular anastomoses, instead of comparing laparoscopic-assisted tubular anastomosis and total laparoscopic triangular anastomosis, to minimise the influence of factors associated with total laparoscopic radical resection. These factors include the small incision, time required to expose the intestinal tube, and so on. In addition, the advantages and disadvantages of the two anastomoses were only analysed according to the anastomotic method used.

When we compared the two anastomotic methods, we found no significant differences in operation time, blood loss, or lymph node dissection. These findings indicate that the two methods both achieve similar quality control standards. The postoperative recovery of flatulence is an objective criterion for determining the recovery of intestinal function. Here, there were no significant between-group differences, suggesting that both methods achieved the same effect on intestinal function recovery. No serious postoperative complications—such as anastomotic leakage or anastomotic stenosis—were observed in either group. Both groups had isolated incidences of anaemia, hypoproteinaemia, and incision infection. Although there were no significant differences between the two groups, intestinal surgery—especially right colon surgery—affected patients’ nutritional status and immunity. Postoperative abdominal haemorrhage occurred in two patients who underwent tubular anastomosis. Haemorrhage is caused by rupture of small mesenteric vessels and is related to insufficient intraoperative haemostasis. There was one case of postoperative abdominal abscess and three cases of chylous leakage in the tubular anastomosis group. Upon closer inspection, we found that these patients had more lymph node metastases. This led to extensive lymph node dissection and greater surgical trauma, which may have been the cause of abdominal abscess and chylous leakage. Anastomotic haemorrhage after triangular anastomosis was related to incomplete postoperative anastomotic suturing and resolved following continuous irrigation and drainage, blood transfusion, and haemostasis. The reason why the cost of hospitalisation was significantly lower in the triangular anastomosis group was showed in Table 3. By analysing the cost of different surgical consumables, we found that for the modified triangular anastomosis two linear staplers are used that costed 4000 RMB and for the tubular anastomosis one circular stapler and one linear stapler are used that costed 8000 RMB. In addition, we also found that the cost of suture in tubular anastomosis was higher than that in modified triangular anastomosis (p < 0.05). However, there were no obvious cost differences of other materials, such as anti-adhesion materials, hemostatic materials between anastomosis method. So we thought different expense of staplers and suture affected total hospitalization expenses in two group.

**Table 3:** The comparison of the cost of different intraoperative consumables in two anastomotic methods

	Modified triangular anastomosis (n = 33)	Tubular anastomosis (n = 59)	Statistical value	P value
Hospitalization expenses(RMB)	49,593 ± 1308	56,548 ± 1166	t = 3.777	0.0003*
Anti-adhesion materials	459.0 ± 67.89	511.0 ± 52.08	t = 0.603	0.5478
Hemostatic materials	1211 ± 165.4	1496 ± 260.4	t = 0.7701	0.4433
Suture lines	1512 ± 261.1	3523 ± 650.9	t = 2.250	0.0269*
Staplers cost	4000	8000		
Other surgical consumables cost	13,090 ± 1271	12,850 ± 846.2	t = 0.1625	0.8713

All fees are in RMB; *p < 0.05

None of the patients enrolled in this study developed an intestinal obstruction. We believe that, for postoperative anastomotic leakage prevention, the tubular anastomosis is more appropriate for treating patients with colon cancer complicated by an intestinal obstruction because of intestinal dilatation, limited blood supply, ectopic flora of intestinal wall, or other factors.

## Conclusion

Laparoscopic tubular anastomosis and triangular anastomosis achieved similar surgical outcomes and had similar rates of postoperative complications. Both achieved acceptable therapeutic effects. Given the equivalent outcomes between the two procedures, the modified triangular procedure may be more a more cost-effective option for clinical application. Given its advantages—including the use of a small incision and quick recovery of intestinal function—continued improvement of laparoscopic radical resection methods for right colon cancer is warranted.

## Data Availability

The datasets analyzed during the current study are available from the corresponding author on reasonable request.

## Abbreviations

CME: Complete mesocolic excision
CVL: Central vascular ligation
